# QuickFigures: A toolkit and ImageJ PlugIn to quickly transform microscope images into scientific figures

**DOI:** 10.1371/journal.pone.0240280

**Published:** 2021-11-09

**Authors:** Gregory Mazo

**Affiliations:** Memorial Sloan-Kettering Cancer Center, New York, New York, United States of America; University of Braunschweig - Institute of Technology, GERMANY

## Abstract

Publications involving fluorescent microscopy images generally contain many panels with split channels, merged images, scale bars and label text. Similar layouts of panels are used when displaying other microscopy images, electron micrographs, photographs, and other images. Assembling and editing these figures with even spacing, consistent font, text position, accurate scale bars, and other features can be tedious and time consuming. In order to save time, I have created a toolset and ImageJ Plugin called QuickFigures. QuickFigures includes many helpful features that streamline the process of creating, aligning, and editing scientific figures. Those features include tools that automatically create split channel figures from a region of interest (“Quick Figure” button and “Inset Tool”), layouts that make it easy to rearrange panels, multiple tools to align objects, and “Figure Format” menu options that help a user ensure that large numbers of figures have consistent appearance. QuickFigures was compared to previous tools by measuring the amount of time needed for a user to create a figure using each software (QuickFigures, OMERO.figure. EZFig, FigureJ and PowerPoint). QuickFigures significantly reduced the amount of time required to create a figure. The toolsets were also compared by checking each software against a list of features. QuickFigures had the most extensive set of features. Therefore, QuickFigures is an advantageous alternative to traditional methods of constructing scientific figures. After a user has saved time by creating their work in QuickFigures, the figures can be exported to a variety of formats including PowerPoint, PDF, SVG, PNG, TIFF and Adobe Illustrator. Export was successfully tested for each file format and object type. Exported objects and text are editable in their target software, making them suitable for sharing with collaborators. The software is free, open source and can be installed easily.

## Introduction

Microscopy intensive scientific manuscripts often include figures with large numbers of fluorescent microscope images often split by channel into separate panels. A major publication can contain over 200 image panels [1]. To create a professional looking publication one must have consistent format within and between figures. However, commercial software do not provide quick ways to split channels and organize panels to generate figures without scores of error prone and repetitive mouse drags. Furthermore, major edits to figures like these are troublesome whether using Adobe Illustrator or Photoshop. For example, cropping or reordering over a dozen identically sized panels in Photoshop without mistakes or accidental changes in panel spacing cannot be done conveniently nor quickly. Although some tools for creating and formatting scientific figures have been created [2, 3], these did not suit our needs because they did not save time on many irksome but important steps. Therefore, I created QuickFigures, a set of tools that 1) can produce figures suitable for any journal format, 2) is user friendly and easy to learn, 3) can save hours of time, 4) can export files for editing in popular software like Adobe Illustrator, Microsoft PowerPoint, and Inkscape 5) is versatile enough to generate any conceivable style, layout, variation or format of figure and, 6) is used as a free PlugIn for ImageJ/FIJI, making installation and adoption easy for researchers already familiar with ImageJ. Here, I explain the resulting software.

## Materials and methods

QuickFigures was written in Java, the same language as ImageJ (using the same version of Java). Computer code was finished using Eclipse IDE Version: 2020–03 (4.15.0) on a Windows 10 system with 2.1 GHz processor 12Gb RAM. Additional testing was performed on a macOS High Sierra with 3.6 GHz processor, 8Gb RAM. QuickFigures uses ImageJ for several key processes including 1) keeping track of an image’s spatial scale and units, 2) the assembly of channels into a merged image based on channel colors and display range, 3) scaling and rotating images, 4) keeping track of channel colors, channel order and metadata about channels (channel names/channel exposure times) and, 5) serializing/storing ImageJ’s multichannel images. Since several complex processes could be performed using ImageJ, this eliminated the need to reimplement existing algorithms. However, the connection between QuickFigures core components and ImageJ was designed such that ImageJ could be replaced by another package if future needs demand it. The user interface for QuickFigures was written using java foundation classes and can function on both Windows 10 and MacOs systems. The “Plot Package” for QuickFigures was written as a separate Java project and designed as an extension to QuickFigures. Although it is possible to write extensions, QuickFigures was written primarily for the convenience of users and not for programmers. The source code for QuickFigures is openly available on GitHub under the Apache License, Version 2.0, allowing for free usage, distribution, and modification (See https://github.com/grishkam/QuickFigures).

### Procedure to measure the fastest time to create a preliminary figure and compare software

To quantify the *minimum* amount of time to create a preliminary figure, a 10-panel split channel figure was made using QuickFigures, EZFig, FigureJ and OMERO.figure. Prior to attempting to measure the time required to create each figure, a user spent a few hours learning each software and practiced making 3–8 figures with each. As a control, the same user practiced making the same figure in PowerPoint. With a timer running, the user created split channel panels, added a second image with split channel panels, aligned panels, added labels (channel label and row label), edited label text, aligned labels, set label font, edited the scale bar, and edited the scale bar’s label. In the case of EZFig, FigureJ and PowerPoint, multichannel images were first cropped, changed into RGB format, and saved as an image sequence using ImageJ. This method was selected because those three lacked either the capacity to display 16-bit multichannel stacks in the same way as ImageJ or lacked convenient ways for a user to visually select a crop area that would be consistent from one image panel to the next. Cropping in ImageJ first also took less time than cropping in PowerPoint, lowering the total time measured. The time spent learning the fastest method to create a figure in each platform was not measured. In the case of OMERO.figure the time needed to upload images to OMERO.server and annotate images was not considered as that would be part of normal workflow prior to making figures.

While studying each software in preparation for the time measurement test, the same user also created a list of features observed in each software or mentioned in the user guide for that software. That list was organized, expanded, and refined to create a more complete comparison between software present in S1 Table. The comparison included four scientific software (including QuickFigures) and two commercial software (Adobe Illustrator and PowerPoint). If a feature appeared to be absent from one software, the user examined the user guides, video tutorials and menus in more detail to be sure. To limit the complexity of the comparison, features were listed in Yes/No format when possible.

### Import and export

The **Bio-Formats Importer** created by the OME consortium is a tool for importing proprietary microscopy file formats into ImageJ [4]. This Importer also reads important metadata regarding spatial scale (needed for scale bars) and channel information (names/channel colors). Assuming that a .zvi, .czi, .lif or other microscopy format file is opened using Bio-Formats, QuickFigures will use the metadata as a basis for the initial channel labels within a figure. To test the accuracy of channel names, a series of example images stained with known markers in known colors were opened manually.

To export figures into PPT, EPS, PDF and SVG file formats, two toolkits created by the Apache Foundation were used as libraries (POI for PowerPoint, https://poi.apache.org/ and Batik for SVG, https://xmlgraphics.apache.org/batik/). Specifically, Batik Version 1.13 and POI Version 4.1.2 were used.

To test the export process, a series of examples containing all types of objects were generated (see source code on GitHub): The examples included multiple images, text items, scale bars, plots, arrows, and other shapes. These examples were designed such that any defect in the export of each particular feature (color, font, line thickness, text angle, object location, line dashes, image panel size and so on) would be visually obvious in the relevant example. To describe a few examples: one test included 25 different kinds of shapes, another included text with Bold, Italic, Strikethrough, Underlined, Superscript, Subscript text at several angles. Yet another test included both Greyscale and Colored image panels. After export, each test file was opened to confirm that each object appeared visually identical in the new software and could be edited. PDF files were opened using Adobe Acrobat, PPT files were opened using PowerPoint, SVG files were opened using Inkscape, PNG files with Microsoft Photos, while EPS and AI files were opened in Adobe Illustrator.

### Other testing

QuickFigures includes scores of objects, windows, menus, and components. Although it is impossible to describe all the testing in a short publication some functions of QuickFigures demand such explanation due to their importance. Of these, accuracy of scale bars is the most important feature.

#### Methodology for scale bar test

Both Tiff files saved with ImageJ and proprietary microscopy formats store information about the physical distance that corresponds to one pixel of an image within saved files (example: 0.1 microns per pixel). Therefore, the proper length of a scale bar may be calculated simply by dividing the desired length (example 10 microns) by that distance (example: For a 100-pixel scale bar of 10 micron). This is the basis for both ImageJ scale bars and QuickFigures scale bars. A user of QuickFigures may also choose to change the size of an image panel in two different ways. One method functions the same as the ‘Scale…’ command in ImageJ and may result in a loss of image quality. The other method does not change a single pixel but changes the panel size and pixel density (no loss in image quality). Regardless of how a user chooses to change the size of image panels, QuickFigures alters the size of scale bars after a user makes any change that affects image panel size and triggers an update to the display. Therefore, tests need to confirm that 1) scale bar sizes are correct and 2) scale bar sizes are updated after changes. For testing purposes, a sequence of images was taken using different objectives on two different Zeiss Observer Microscopes. The pixel size displayed in the software was recorded (even if displayed in rounded form) and the expected scale bar sizes were calculated (using both calculator and Excel). An automated JUnit test was written to confirm that the size of scale bars in QuickFigures matched the expected length that was calculated (see source code on GitHub). Those same JUnit tests also checked whether the size of the scale bar appropriately changed when scale factor or panel size was changed. As an additional (manual) test, a scale bar was created manually using the scale bar tool in ImageJ and visually compared to the QuickFigures scale bar to confirm that the two scale bars were the same size. Image panels were subsequently resized to make sure that the scale bar objects appropriately changed size. Next, the units and spatial scale of the ImageJ image was changed manually and the user checked to make sure that the scale bar in QuickFigures changed appropriately.

To test the figure format menu commands (a complex feature) both partly automated and fully manual tests were done. For the manual tests, every possible type of figure edit was manually performed on a single figure and that figure’s format was stored. Specifically, the details changed included the image scale factor (changed by a factor of 2), label font (to a visibly larger and distinct font), label position (to a different location), panel spacing (3-fold larger), scale bar style, text angle (from 0 to 45 degrees), and merge label style (from black ‘merge’ to multiple colors). Each of these changes were made such that the difference would be visually striking for each object. That stored format was then applied to a new target figure (that started with a different appearance). A human tester then visually confirmed that each target figure had been altered to match the form of the stored figure format. (see S1 Video for demonstration of figure format menu).

#### Practical Test

The ultimate test for QuickFigures is usability by an ordinary researcher. Since creation of an early version, the developer has used QuickFigures constantly for preparation of figures for presentations and publications. Every format, layout and style of figure was generated for various scientific projects. This was done on both Windows 10 and Mac operating systems. This long pre-release period provided time to detect, understand and fix many irregularities, bugs, error messages and limitations. Extensive revisions and additional bug fixes were performed in response to reviewer comments. After revisions, QuickFigures was shared with three colleagues who were asked to use the software for their own purposes and report whether they were able to install QuickFigures on their own, understand the tutorials, and create their figures without difficulty. Users were also asked to report any bugs. For additional feedback, a similar request was posted to the wider ImageJ community using an online ImageJ forum.

#### Installation

For users of Fiji or ImageJ2, QuickFigures can be installed using the ImageJ ‘Update…’ command. Users only need to choose QuickFigures from the list of ImageJ update sites. Fiji or ImageJ2 is strongly recommended. For users of standard ImageJ, QuickFigures may be downloaded from GitHub and can be installed into ImageJ simply by placing the file into plugins folder of ImageJ. Users of standard ImageJ will need to follow instructions in user guide if they need to install export packages.

## Results

### Basic use: Creating a figure

**Fig 1:**
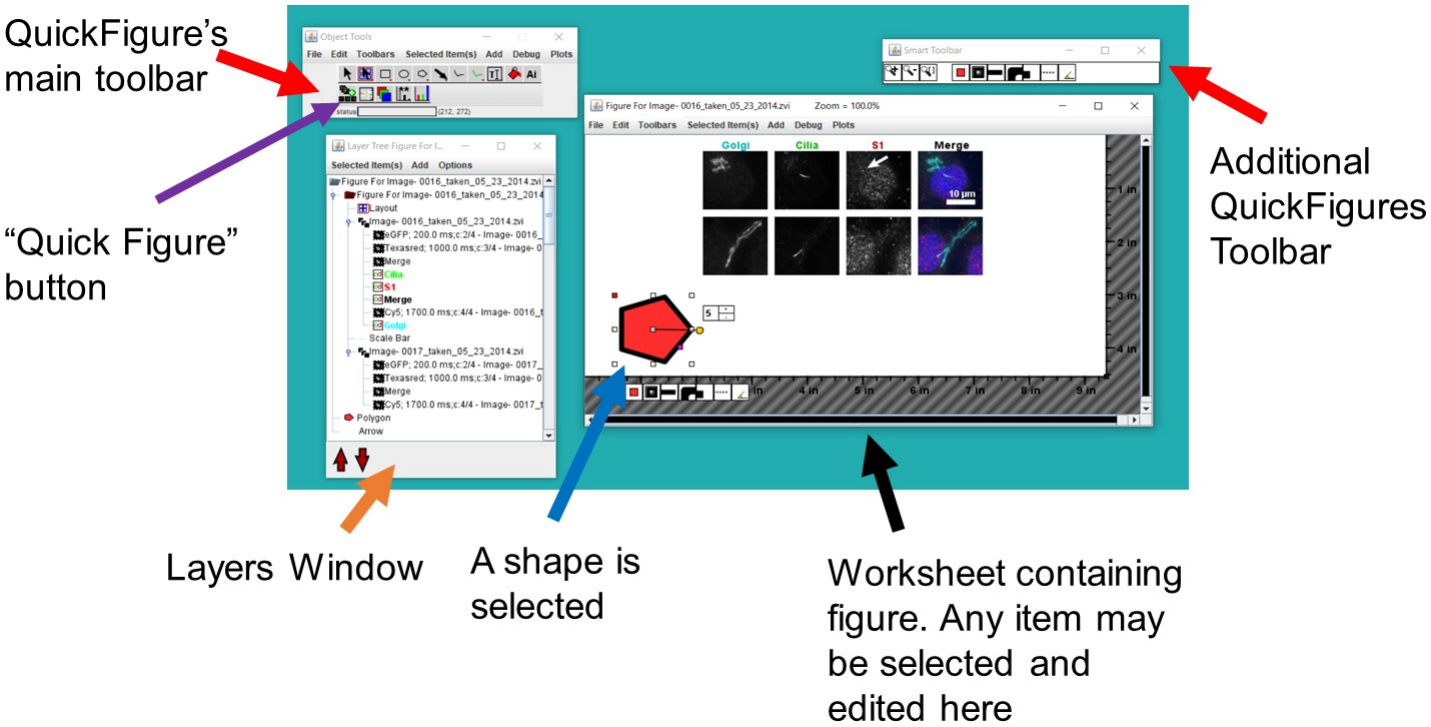
The QuickFigures user interface. An open figure is displayed. Black arrow points to the worksheet window where nearly all edits are performed with the mouse. Red arrows indicate toolbars. The main toolbar on the left contains drawing tools, the ‘Quick Figure’ tool and the ‘Inset’ tool indicated by violet arrow. A single pentagon is drawn and selected to demonstrate drawing and object selection. All objects can be selected and edited. The layers window displays a list of layers and objects (orange arrow).

A screenshot showing the user interface of QuickFigures is shown in Fig 1. QuickFigures contains unique toolbars (Fig 1, see red arrows). Users can create a figure simply by first opening a multidimensional image stack in ImageJ, and then clicking the “Quick Figure” button that is visible on the main toolbar of QuickFigures (Fig 1, see purple arrow). This one click creates a figure with a series of split channel images, a merged image, channel labels and scale bar. The user can edit every part of this figure which is shown in the worksheet window (Fig 1, black arrow). Any number of images may be added by simple drag/drop of multichannel image files onto the worksheet window. In the case of large images, users may be prompted to select a rectangular area of interest that can be edited later. Automatically generated figures consist of many separate objects organized into layers (Fig 1, Orange arrow indicates layers window that shows the layers with list of objects).

### Other advantages provided by QuickFigures

QuickFigures automates and facilitates mundane tasks that arise naturally during the editing process. Changes in panel spacing or panel order can be quickly applied to figures with dozens of panels. Editable scale bars of correct size are generated based on the meta-data within image files and the size of image panels. When one resizes an image panel, scale bars appropriately adjust to the correct size (See Methods section for details about how the accuracy of generated scale bar functions were tested). Label text signifying each channel is colored and aligned automatically. When microscopy images are imported into ImageJ using Bio-Formats, QuickFigures’ generates colored channel labels that are consistent with the channel names given by Bio-Formats (DAPI, eGFP, mCherry for example). Image resolution can be changed as needed. When a user adjusts the display range (Min/Max) of one channel, QuickFigures alters every image panel that contains the channel (Merge and Split) ensuring consistency of all adjustments across groups of image panels. Dozens of images can be cropped to the same size and positioned automatically. A series of labels with consistent alignment can be created easily. Importantly, a user can set a “default template” for figures to ensure that a single consistent format is automatically applied to newly created figures (Templates can also be applied to existing figures using the “Figure Format” submenu). A special tool called the “Inset Tool” also automates several steps. When the user draws regions of interest on an image panel using the “Inset Tool”, several additional image panels showing the region of interest at a higher level of magnification are automatically generated. A user can edit the regions shown by the additional panels simply by editing the region of interest or right clicking on the region of interest to display its popup menu. In aggregate, several slow, or complex tasks are made fast and simple.

### Editing figures

**Fig 2:**
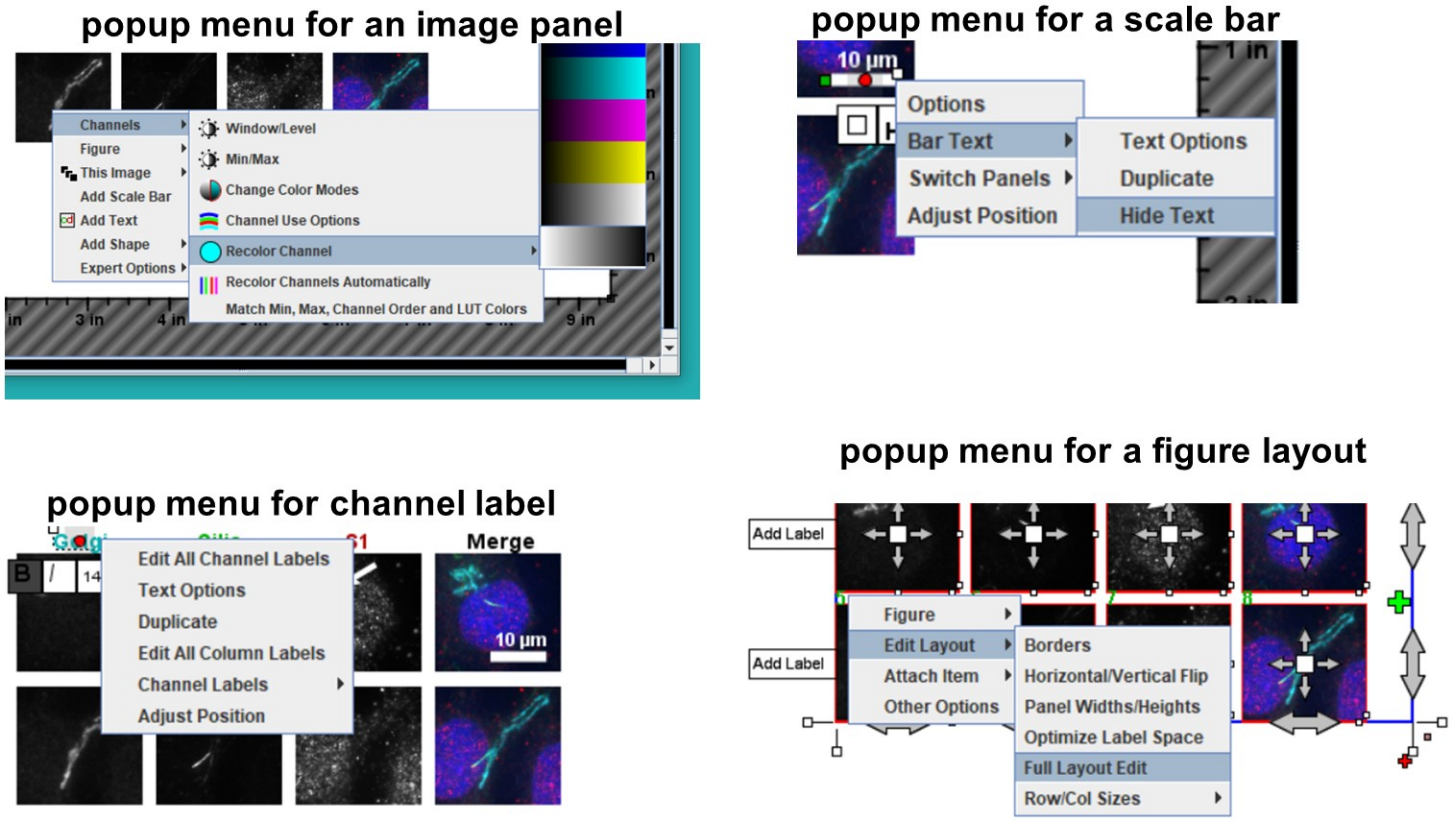
Popup menus are specific for different types of objects. Screenshot shows the popup menus for three types of objects: an image panel that is part of a figure, a scale bar, a layout and a channel label.

Every item in QuickFigures is an editable object that can be clicked on, moved, resized, rotated, hidden, deleted, aligned, edited, or duplicated (Fig 1, blue arrow indicates a selected item). A simplistic set of menus and toolbars allows the user to add an unlimited number of items such as text, images, drawings, arrows, layers, plots, shapes, ordinary images, and additional multichannel images. Right clicking on any item reveals a popup menu specific to the clicked item (Fig 2). Since each popup menu contains options related to specific kind of object, a user can easily find the option that is needed.

**Fig 3:**
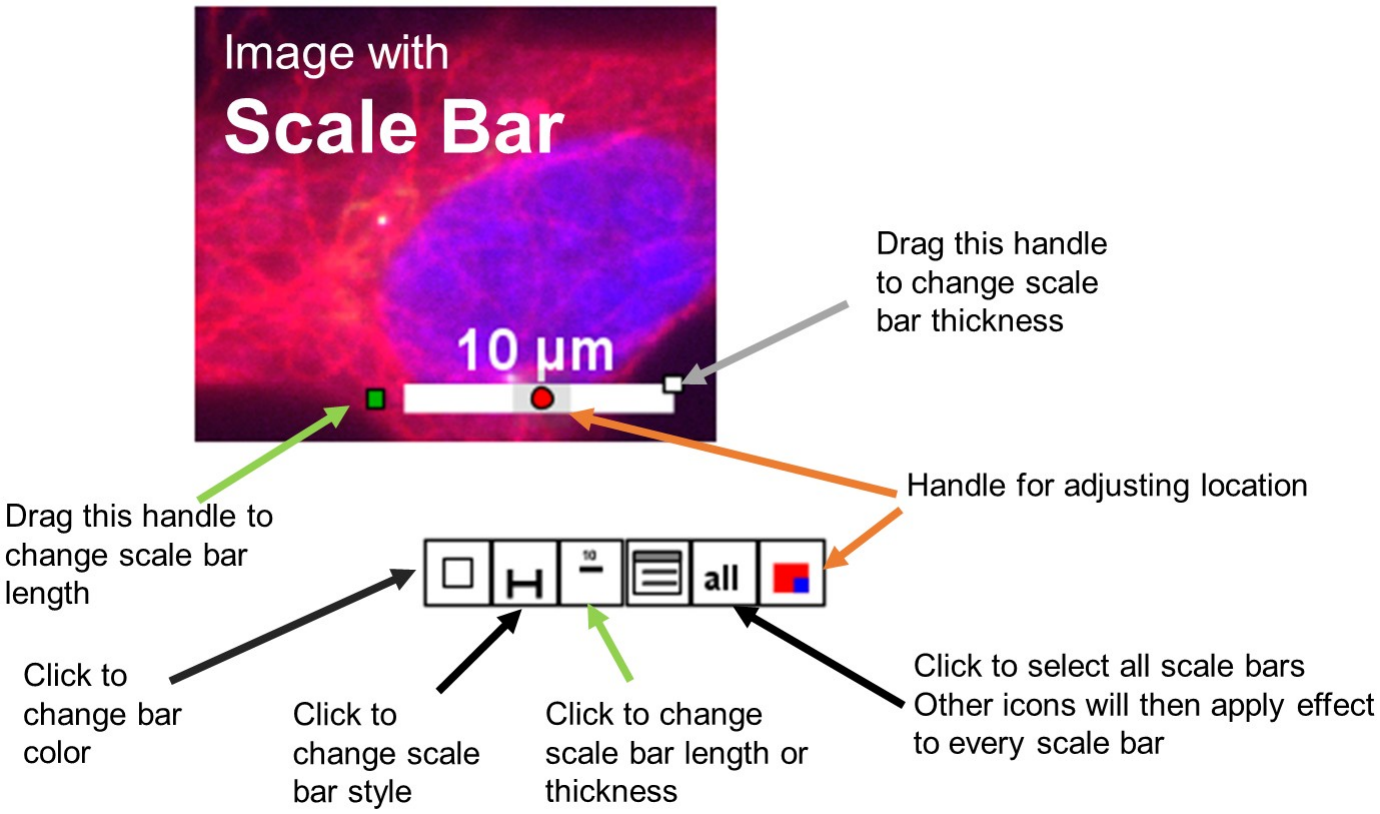
Handles and shortcuts are available when an item is selected. Screenshot shows an example of a selected object. Several icons and handles are visible when the object is selected. User may change the length of the scale bar by dragging a handle or clicking an icon for a menu (green arrows). Similarly, the user may fine tune the location of an object with the handles indicated by orange arrows. Other options are also available (black and grey arrows). Descriptions and diagrams for each major object type are shown in user manual.

**Fig 4:**
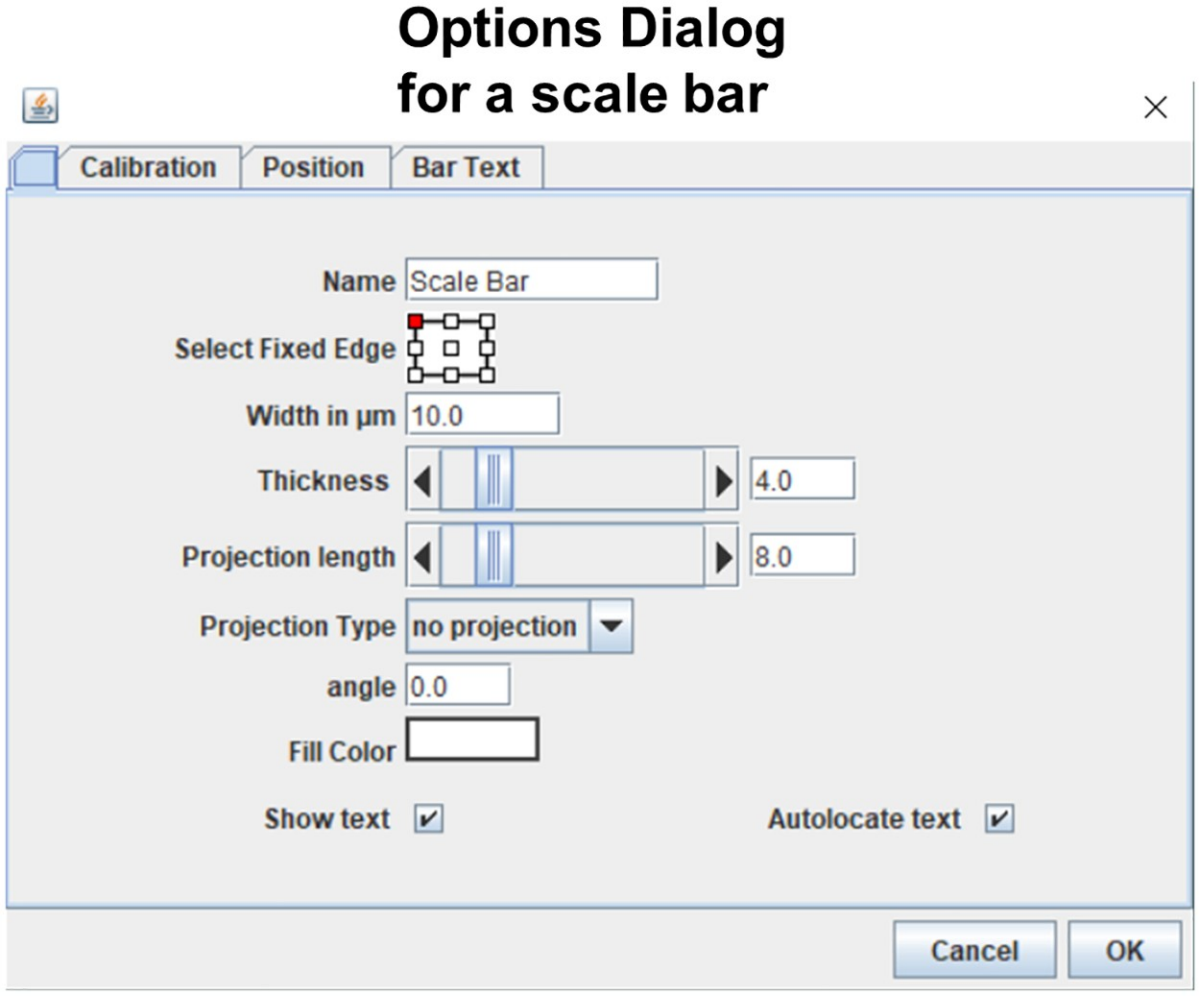
Objects can be edited with dialogs. Screenshot shows the dialog for one item (scale bar). Every option related to scale bar editing is listed by name in the dialog.

A user may click on an item to select it and then edit that item by dragging the handles. Figure layouts can also be edited by clicking and dragging handles. When an item is selected, a set of icons appears below the item (analogous to the mini toolbars in office applications). Users may click on those icons to perform any type of edit (See scale bar example in Fig 3). Alternatively, users may double click an object to open a dialog window in which options specific for the clicked object are listed by name (Fig 4). In either case a user can easily find the option that is needed to perform the desired edit.

As a guide to help first time users, I created an online user manual and a series of video tutorials. The basic features of QuickFigures are meant to be easily comprehended by any researcher. New users may learn the most important features and start using QuickFigures in under 10 minutes by watching a single video (S1 Video). Users who want to learn more details about how to use QuickFigures may watch the two other tutorials (S2 and S3 Videos). Also available on YouTube, the series of videos provides short explanations of the features and clearly demonstrates the usefulness of QuickFigures. In contrast, the user guide contains screenshots and detailed descriptions of every feature (available on GitHub). In summary, the specialized features of QuickFigures require only minutes of explanation while saving time and providing convenience.

### Export

After a user has created work in QuickFigures, it is necessary to export figures to other file formats for further editing, presentation, distribution, or publication. QuickFigures can export files into several file formats including TIF, PNG, PDF, EPS, PowerPoint (PPT), and Scalable vector graphic (SVG), a format that can be opened by many popular software (including Inkscape and Adobe Illustrator). Users with Adobe Illustrator installed will also have the option to generate the same figure in Adobe Illustrator, which then saves it in Adobe Illustrator format (.AI). When objects are exported into vector formats, each item is a separate editable object and therefore those exported figures are suitable for additional editing. This is particularly useful when working with teams of colleagues who may want to receive files in their own favored platform. The export functions were tested on a series of images containing every possible type of object (See Materials and methods). In each case, exported figures visually resembled originals. However, since not every font nor every line dash is available for every possible file format, it is not always possible for exported files to be 100% identical to originals. If a user chooses an exotic font that is not available in the target export format nor the target software, the exported text will (obviously) not be shown with the desired font. Some fonts may also appear differently in different software. In addition, dashed lines in PowerPoint do not function the same way as dashed lines in other formats and look different. Except for these caveats, user exported figures will appear essentially identical to the originals created in QuickFigures.

### Comparison to existing tools

**Fig 5:**
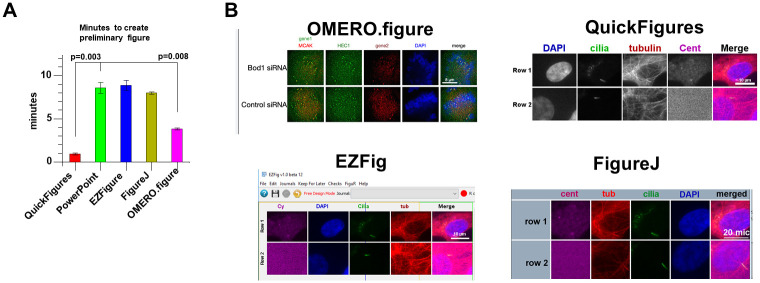
Less time is required to create a figure using QuickFigures. (A) Plot shows the time required to create a figure with each software. Error bars depict standard deviation. 3 replicates of each time measurement were done. (B) Images of figures created during the time measurement test are shown.

Over the past decade, a few other toolsets that assist in the process of creating scientific figures with images have been created. These include ImageJ Plugins such as FigureJ, ScientiFig, EZFig (a newer version of ScientiFig) as well as OMERO.figure, a web-based tool. While each of these tools introduced specialized features such as layouts and automatic alignment of labels, they generally lacked the flexibility, easy editing, and basic text/drawing features present in general purpose applications such as Adobe Illustrator, Inkscape or PowerPoint. Although QuickFigures was designed with many novel and unique features, QuickFigures also contains most of the specialized scientific features seen in other packages. Furthermore, QuickFigures was designed to mimic the flexibility of general-purpose drawing software and has a broader diversity of export options than comparable toolsets. To perform a detailed comparison between QuickFigures and alternative figure building software, I carefully examined each software and created a list of more than 100 traits that differed between them. To summarize, QuickFigures automates more steps than other tools (to save more time), is more flexible and contains a larger variety of features/options. See S1 Table for detailed descriptions of the advantages provided by each feature. Of the features that were scored, QuickFigures contains 89% compared to <60% for the other toolsets (S1 Table), highlighting a larger number of features. This comparison also covers excellent features that are not available in QuickFigures such as a feature to check objects against the format requirements of known journals (present in EZFig) or the maximal intensity projection feature (available in OMERO.figure). In addition to the qualitative differences, the amount of time saved differed between toolsets. Features within QuickFigures such as the ‘Quick Figure’ button are designed to reduce the time required to create a split channel figure (like that shown in Fig 1). The time required for a user with detailed understanding of each software to create a figure was measured. Using QuickFigures, one can generate a preliminary figure in about one minute, significantly less than any alternative package (Fig 5). Although the time spent learning each software was not measured, the user interfaces for QuickFigures and OMERO.figure are somewhat less complex to learn than the others.

### Practical testing and user feedback

During the revision of this manuscript, three colleagues were asked to evaluate QuickFigures and provide feedback. In this ‘practical test’, users confirmed that they were able to understand tutorials and use QuickFigures to make their own figures. Only one of the three identified a problem (that was fixed). Subsequently, more requests for feedback were posted to an ImageJ forum. Feedback at this late stage consisted largely of ideas for new features that could improve QuickFigures. Some suggestions (such as an option to change that interpolation method is used for scaling) have already been implemented. Other feedback from the community consisted of questions about existing functions (answers are in user guide). The help of this community (and peer reviewers) resulted in a better-quality toolset than would otherwise be possible. Additional ideas from users will be added to QuickFigures in the future. These include a menu option to add ‘A, B, C…’ labels to a group of selected objects and another menu option to ‘mirror’ the same region of interest onto multiple image panels. Depending on user feedback, more additions, and improvements to QuickFigures may be done.

## Discussion

Normally, scientific figures are created using a general-purpose drawing software such as PowerPoint, Adobe Photoshop, Adobe Illustrator or Inkscape. Drawing applications are versatile, powerful, have well designed user interfaces and are familiar to users. However, these applications lack specialized features for production of scientific figures. Therefore, building scientific figures in such applications becomes a time-consuming process. While including many time-saving features, QuickFigures was designed to feel familiar and logical to anyone who has used PowerPoint, Photoshop, Illustrator, or any commercial software. Therefore, researchers only require introduction to the specialized features to understand the software. Other tools for preparation of scientific figures have become available in the past several years. Users of ImageJ can install EZFig, ScientiFig (an older version of EZFig) or FigureJ. Users at institutions using OMERO.server and OMERO.web can use OMERO.figure, an extremely well-designed web-based tool. While both QuickFigures and OMERO.figure provide a wide range of features specifically for fluorescent microscopy images, FigureJ and EZFig focus more on layouts, alignment, and panel arrangement. QuickFigures differs from all other toolsets in important ways. QuickFigures includes several features that are not available elsewhere. Tools such as the ‘Quick Figure’ tool, the “Inset Tool” and the ‘Figure Format’ menu automate several different steps (See S1 Video). These three tools do not have truly comparable substitutes. Although both FigureJ and EZFig have layouts that assist with panel alignment and rearrangements, the layouts in QuickFigures function differently from the layouts in other software. In QuickFigures more than one layout can be created in a single figure, layouts may be moved to any location, image panels are not permanently attached to layouts, and layouts can be aligned to each other making the layout functionality in QuickFigures more flexible. Flexible layouts help users create more complex figures. QuickFigures is more versatile with more options for any given feature and a user interface as simple as a general-purpose drawing software. QuickFigures also includes a large number of user options for editing figures that show multichannel fluorescent images, or stacks of images. In summary, QuickFigures can help many researchers save hours of time that would otherwise be spent performing tedious modifications to figures. That time will certainly be put to more productive use.

## Supporting information

S1 Table Detailed comparison of toolsets. Table shows list of software characteristics that differ between QuickFigures and other software along with other useful information. Each trait was scored in a Yes/No manner and total scores are shown at the bottom of the table.
(XLSX)

S1 Video Tutorial video 1, introduction video. Video demonstrates the ‘Quick Figure’ tool, the “Inset Tool”, the “Figure Format” menu, layouts, and other features.
(MOV)

S2 Video Editing figures. Video demonstrates how to edit several types of figures.
(MOV)

S3 Video Figure format and other features. Video demonstrates how to change the default template for figures and several other features.
(MOV)

S1 Links (DOCX)
