# Supplementary material for: QuickFigures: A toolkit and ImageJ PlugIn to quickly transform microscope images into scientific figures
Source: PLoS One. 2021 Nov 9;16(11):e0240280. doi: 10.1371/journal.pone.0240280 (PMC8577749; doi:10.1371/journal.pone.0240280)
Supplement: S1 Links — (DOCX) [file pone.0240280.s005.docx]

**Links**

**GitHub page with source code:**

[**https://github.com/grishkam/QuickFigures**](https://github.com/grishkam/QuickFigures)

**User Guide:**

[**https://github.com/grishkam/QuickFigures/blob/master/UserGuide/User%20Guide.md**](https://github.com/grishkam/QuickFigures/blob/master/UserGuide/User%20Guide.md)

**Tutorial Video Playlist:**

**https://www.youtube.com/watch?v=9Crg-FAOHmc&list=PLM5I73cb55tDX4XCjKGK-Jm3-tJsUb7qm**
